# Establishment of an immortalized intestinal epithelial cell line from tree shrews by lentivirus-mediated *hTERT* gene transduction

**DOI:** 10.1007/s10616-018-0270-0

**Published:** 2019-01-02

**Authors:** Bowen Yin, Qingkai Song, Lingxia Chen, Xiaofei Li, Yuanyuan Han, Xuan Wang, Jiejie Dai, Xiaomei Sun

**Affiliations:** Center of Tree Shrew Germplasm Resources, Institute of Medical Biology, The Chinese Academy of Medical Science, Peking Union Medical College, Jiaoling Road 935, Kunming, 650118 China

**Keywords:** Immortalized, Intestinal epithelial cell line, Tree shrews, Lentivirus-mediated *hTERT* gene

## Abstract

The intestinal epithelium has an average lifespan of 4–5 days. Normally, primary intestinal epithelial cells can be cultured for about 15 days in vitro. The aim of this study was to explore methods to isolate and immortalize intestinal epithelial cells (IECs) of tree shrews in order to establish a new resource of experimental material and to provide a cell model for drug development and infection mechanism research. Tissue from the small intestine of tree shrews was digested with collagenase XI, neutral protease I, and dithiothreitol. The human telomerase reverse transcriptase gene (*hTERT*) was transferred into tree shrew IECs using the pHBLV-CMVIE-ZsGreen-Puro vector. The level of hTERT mRNA was detected by quantitative reverse transcription polymerase chain reaction. Immunofluorescence and western blot assays were performed to detect biochemical markers of IECs. The micromorphology of cells was observed with electron microscopy. We then conducted experiments to assess proliferative activity and analyze the karyotype of isolated cells. The results showed the immortalized cell line that we established and screened, maintained the characteristics and biochemical markers of primary IECs. Our results showed that the cell line we established can be considered an alternative cell model for intestinal drug research and for studies on intestinal infection and cell signaling.

**Electronic supplementary material:**

The online version of this article (10.1007/s10616-018-0270-0) contains supplementary material, which is available to authorized users.

## Introduction

Tree shrews (*Tupaia belangeri chinesis*) are small mammals that are close relatives of primates and show a high degree of similarity to humans in their anatomy and physiology, neural development, and responses to viral infection and psychological stress (Xu et al. 2013). Compared with other non-primate mammals, tree shrews have a high brain-to-body mass ratio (Xu et al. 2012). Other characteristics, such as their small size, short breeding cycle, and low food and research costs make the tree shrew an ideal animal model.

The intestinal epithelium forms a natural physical barrier and maintains the structural stability of the intestine. However, the intestinal tract is exposed to a large number of antigens (Martini et al. 2017) and gut inflammation can easily disrupt the intestinal epithelium. Recent studies have revealed that the intestinal microenvironment is complex and that dysbiosis can result in various diseases (Tanaka and Nakayama 2017), including inflammatory bowel disease (Christopher et al. 2015), irritable bowel syndrome (Codling et al. 2010), obesity (Karimi et al. 2015), allergies (Lynch and Boushey 2016), autoimmune diseases, and brain disorders (Wang and Kasper 2014). Models of the intestinal epithelium are important for understanding these diseases. However, long-term in vitro culture of primary intestinal epithelial cells (IECs) is difficult because the epithelium is completely renewed every 4–5 days (van der Flier and Clevers 2009). Additionally, IECs are highly sensitive to anoikis, a form of programmed cell death caused by epithelial cell and extracellular matrix disorders. Therefore, better research models are needed to study IECs.

Telomeres are the repeating nucleotide sequence regions at the ends of chromosomes that protect them from deterioration or fusion with adjacent chromosomes (Witzany 2008). During the process of chromosome replication, DNA polymerase does not extend to the end of the chromosome, causing the ends to shorten after each DNA replication cycle. As telomeres become shorter they reach the theoretical Hayflick limit around 50–70 cycles of cell division, resulting in cell death (Hayflick^1965^). Telomerase is a polyprotein complex made up of a telomerase reverse transcriptase (TERT) catalytic subunit and a telomerase-associated RNA template that adds DNA repeats to the end of the chromosome to prevent telomere shortening (Blackburn 2001). However, human TERT (hTERT) is only expressed in telomerase-positive cells and, shortly after birth, telomerase activity ceases in most normal human tissues. Therefore, *hTERT* gene transfer has been used as a means of immortalizing cells, including human retinal epithelial cells (Bodnar et al.^1998^) and porcine small intestinal epithelial cells (Wang et al. 2014).

In this study, we successfully used a lentivirus-mediated method to infect tree shrew primary IECs with the *hTERT* gene to create an immortalized cell line we called TIEC.

## Materials and methods

### Animals and reagents

Two-day-old neonatal tree shrews were reared in an environment with a temperature of 16–28 °C, 40–70% humidity, noise levels ≤ 60 dB, and light intensity levels of 1001–1501×. Experimental animals were obtained from the Center of Tree Shrew Germplasm Resources, Institute of Medical Biology, Chinese Academy of Medical Science and Peking Union Medical College with the production license number SCXK (Yunnan) K2013-0001. The Institutional Animal Care and Welfare Committee of the Institute of Medical Biology, Chinese Academy of Medical Sciences and Peking Union Medical College approved the present study, and all procedures were performed according to ethical standards and practices.

The following reagents were used: dithiothreitol (DTT) (Biosharp), collagenase XI (catalog # C7657, Sigma-Aldrich), insulin-transferrin-selenium (ITS-G; Life), epidermal growth factor (EGF; PeproTech), neutral protease I (Solarbio), D-sorbitol (Biosharp), pHBLV-CMVIE-ZsGreen-Puro vector (Shenggong Engineering Co., Ltd.), *Escherichia coli* DH5α (Invitrogen), trypsin (Thermo), Dulbecco’s Modified Eagle’s Medium (DMEM; Thermo), phosphate buffered saline (PBS; HyClone), TaKaRa One Step PrimeScript RT-PCR Kit (Perfect Real Time) (Dalian Bao Bioengineering Co., Ltd.), RNeasy Mini Kit (Qiagen), PrimeScript II 1st Strand cDNA Synthesis Kit (Dalian Bao Bioengineering Co., Ltd.), Golden Green Mix (TsingKe), rabbit anti-cytokeratin 18 antibody (Abcam), anti-occludin antibody (Abcam), anti-β-actin antibody (Abcam), and puromycin (Solarbio).

### Isolation and culture of tree shrew intestinal epithelial cells

Two-day-old tree shrews were euthanized by injecting an overdose of pentobarbital sodium. The small intestine was collected and prepared by removing the longitudinal muscle layer and washing with ice-cold Hank’s Balanced Salt Solution (HBSS) wash solution containing 100 U penicillin, 100 μg/mL streptomycin, 25 μg/mL gentamycin, and 0.5 mM DTT in Mg^2+^- and Ca^2+^-free HBSS (Graves et al. 2014). The tissue was cut into small pieces and washed several times with HBSS wash solution until the supernatant was clear. The contents were allowed to settle for 10 min and the supernatant was discarded. The remaining tissue was placed in 10 mL of digestion buffer containing 1% v/v fetal bovine serum (FBS), 75 U/mL collagenase XI, 20 μg/mL dispase neutral protease II, and 0.5 mM DTT in DMEM. The tissue was digested in a 37 °C incubator with shaking at 180 rpm for 2 h. A 10-mL dispersion solution (DMEM containing 2% w/v D-sorbitol) was then added to the digestion mixture and the tissue was disassociated by repeated pipetting. Remaining tissue debris was discarded and the supernatant containing proliferative crypts was centrifuged at 200×*g* for 8 min. Cell pellets were washed twice in DMEM (high glucose) and resuspended in DMEM (high glucose) containing 10 mM HEPES, 100 U/mL penicillin, 100 mg/mL streptomycin, 10 ng/mL EGF, 1% ITS, and 2% FBS and cultured at 37 °C and 5% CO_2_. Media were changed every 3 days. FBS was adjusted to 10% when cells reached confluence after 10–12 days.

### Construction of lentiviral vector

pHBLV-CMVIE-ZsGreen-Puro containing a ZsGreen gene and a puromycin resistance gene was selected as the vector for the exogenous *hTERT* gene. Primers were designed to amplify the *hTERT* gene sequence provided by the National Center for Biotechnology Information (NCBI) and EcoRI and XbaI restriction sites were added to the 5′ ends of the forward and reverse primers to create primers hTERT-EcoRI/XbaI-F:5′ -ggatctatttccggtgaattcgccaccATGCCGCGCGCTCCCCGCT-3′ and hTERT-EcoRI/XbaI-R: 5′-ggatccgcggccgcttctaga GTCCAGGATGGTCTTGAAGT-3′. The amplified *hTERT* gene and the vector were each cut with EcoRI and XbaI and joined by T4 ligase (Thermo). The constructed lentivirus vector was transformed into *E. coli* DH5α to amplify the plasmid. Three plasmids, PSPAX2, pMD2G, and pHBLV-CMVIE-ZsGreen-Puro, were then co-transfected into 293T cells to package the lentivirus. The culture supernatant from these cells was collected 48 h and 72 h after transfection and ultracentrifuged at 2000×*g* for 10 min at 4 °C. Cell debris was discarded and the supernatant was transferred to a new tube and ultracentrifuged at 82,700×*g* for 120 min at 4 °C. Lentivirus was aliquoted into sterilized tubes and stored at − 80 °C.

The titer of virus was determined by the dilution and counting method. 293T cells were plated in 96-well plates at a density of approximately 1 × 10^5^/mL (100 μL/well) and cultured at 37 °C and 5% CO_2_. The virus solution was diluted three-fold and 10 μL of each dilution was added to the 293T cells. Three days post-infection, wells that contained cells whose percentage of fluorescence was between 10 and 30% were used to calculate the virus titer using the following equation: titer (TU/mL) = cell number × fluorescence ratio × multiplicity of infection (MOI; 1) × virus dilution factor × 10^3^.

### Transduction and screening

IECs passaged to the third generation were plated onto 24-well plates at a density of 1 × 10^4^ cells/mL. The media were discarded and 250 μL of cell maintenance media (10 mM HEPES, 100 U/mL penicillin, 100 mg/mL streptomycin, 10 ng/mL EGF, and 1% ITS) were added. Cells were infected with lentivirus at an MOI of 10 and cultured at 37 °C. The percent fluorescence was determined with a fluorescence microscope 72 h post-infection.

To screen cells, 5 μg/mL puromycin was added to both experimental and control cells. When control cells were completely killed with 5 μg/mL puromycin 2.5 μg/mL puromycin was added to the corresponding experimental cells. Cells were then digested with trypsin and diluted to 10 cells/mL in complete media and single cells (0.1 mL) were seeded into 96-well plates. Wells that contained only one cell, as observed by microscopy, were marked and incubated in a 37 °C incubator to form monoclonal cells.

### hTERT gene expression analysis by quantitative reverse transcription polymerase chain reaction (qRT-PCR)

RNA was extracted from transduced cells using an RNeasy Mini Kit (Qiagen) and reverse transcribed into cDNA using the TaKaRa One Step PrimeScript RT-PCR Kit (Perfect Real Time) (Dalian Bao Bioengineering Co., Ltd.) according to the manufacturer’s protocol. The following *hTERT* gene primers were designed based on the *hTERT* gene sequence: F: 5′-TCCGAGGTGTCCCTGAGTAT-3′ and R: 5′-TGACACTTCAGCCGCAAGA-3′. qRT-PCR was carried out using a 2 × T5 Fast qPCR Mix (SYBR Green I) kit (TsingKE). The reaction contained 10 μL 2 × T5 Fast qPCR Mix, 0.8 μL each upstream and downstream primer, 50 × ROX Reference Dye I/II, and 0.4 μL cDNA (< 100 ng). The following conditions were used for the qRT-PCR reaction: 95 °C for 1 min; 40 cycles of 95 °C for 10 s, 55 °C for 5 s, and 72 °C for 15 s; and fluorescence signal acquisition at 72 °C.

### Immunofluorescence microscopy

Cells were fixed in 4% paraformaldehyde for 30 min at room temperature. Cells were then washed three times with PBS, permeabilized with 0.1% Triton X-100 for 20 min at room temperature, and blocked with 1% bovine serum albumin for 30 min. Cells were immunolabeled with anti-cytokeratin 18 antibodies (1:500) overnight at 4 °C. Cells were then washed with PBS and incubated with goat anti-mouse IgG antibody (Abcam) at room temperature for 1 h. After washing three times in PBS, cells were incubated with 4′,6-diamidino-2-phenylindole (DAPI) diluted 1:500 in PBS for 5 min and imaged using fluorescence microscopy.

### Western blotting

Once confluent, cells grown in six-well plates were rinsed with PBS. Cell lysis buffer containing 1.0 mmol/L phenylmethanesulfonyl fluoride was added and the mixture was boiled for 10 min. Proteins were separated with sodium dodecyl sulfate polyacrylamide gel electrophoresis on a 12% gel, transferred to a polyvinylidene fluoride (PVDF) membrane at 15 V for 1 h, and washed three times with PBS. The PVDF membranes were blocked with skim milk at room temperature for 1 h and washed three times with PBS containing 0.5% Tween 20 (PBST). PVDF membranes were incubated overnight with anti-β-actin antibody or the primary antibodies anti-cytokeratin 18 and anti-occludin diluted in skim milk at 4 °C and washed three times with PBST. Membranes were then incubated with secondary antibodies (1:5000) for 1 h at room temperature, washed three times with PBST, and photographed using a gel imager (Bio-Rad).

### Transmission electron microscopy

Cells were centrifuged, fixed with 2.5% glutaraldehyde, dehydrated, embedded, and sectioned. The ultrastructure of cells was observed using a transmission electron microscope following the protocol of Geens and Niewold (2011).

### Cell growth curves

Primary tree shrew intestinal epithelial cells (pTIECs) and transduced cells were plated onto 24-well plates at 1 × 10^4^ cells per well and incubated at 37 °C and 5% CO2. One well of cells was digested with 0.25% trypsin every 24 h and transferred to a 1.5 mL centrifuge tube. Cells were counted using a hand-held cell counter. Experiments were conducted in triplicate and mean values were plotted for growth curves. Differences between groups were examined for statistical significance using the Student’s *t* test. *P* < 0.05 was considered as statistically significant.

### Optimal FBS concentration determination

Cells were seeded into 96-well plates at 2 × 10^3^ cells per well and incubated for 36 h at 37 °C in complete media containing 0, 2, 5, 10, or 20% FBS. Cell proliferation was measured using the WST-1 Cell Proliferation and Cytotoxicity Assay Kit (Beyotime) and mean values were plotted using histograms. Experiments were conducted in triplicate. Differences between groups were examined for statistical significance using the Student’s* t*-test.

### Karyotype analysis

Cells were washed with PBS and centrifuged at 1500 rpm for 5 min. The supernatant was discarded and cells were washed with PBS for 1 min and centrifuged at 300×*g* for 5 min. The supernatant was discarded and the cell pellet was resuspended in 75 mmol/L KCl and allowed to settle for 8 min at room temperature. One drop of methanol:glacial acetic acid (3:1) fixative was added and the sample was centrifuged at 300×*g* at 4 °C. The supernatant was discarded and 1 mL fixative was added to the sample. The sample was allowed to settle for 30 min at 4 °C, resuspended, and centrifuged at 300×*g* at 4 °C. The supernatant was discarded and 500 μL fixative was added to the sample. The sample was resuspended, dropped onto a clean slide precooled to 4 °C, and air dried. Samples were stained with Giemsa dye for 10 min and observed under a microscope.

## Results

### Monoclonal TIEC screening

**Fig. 1:**
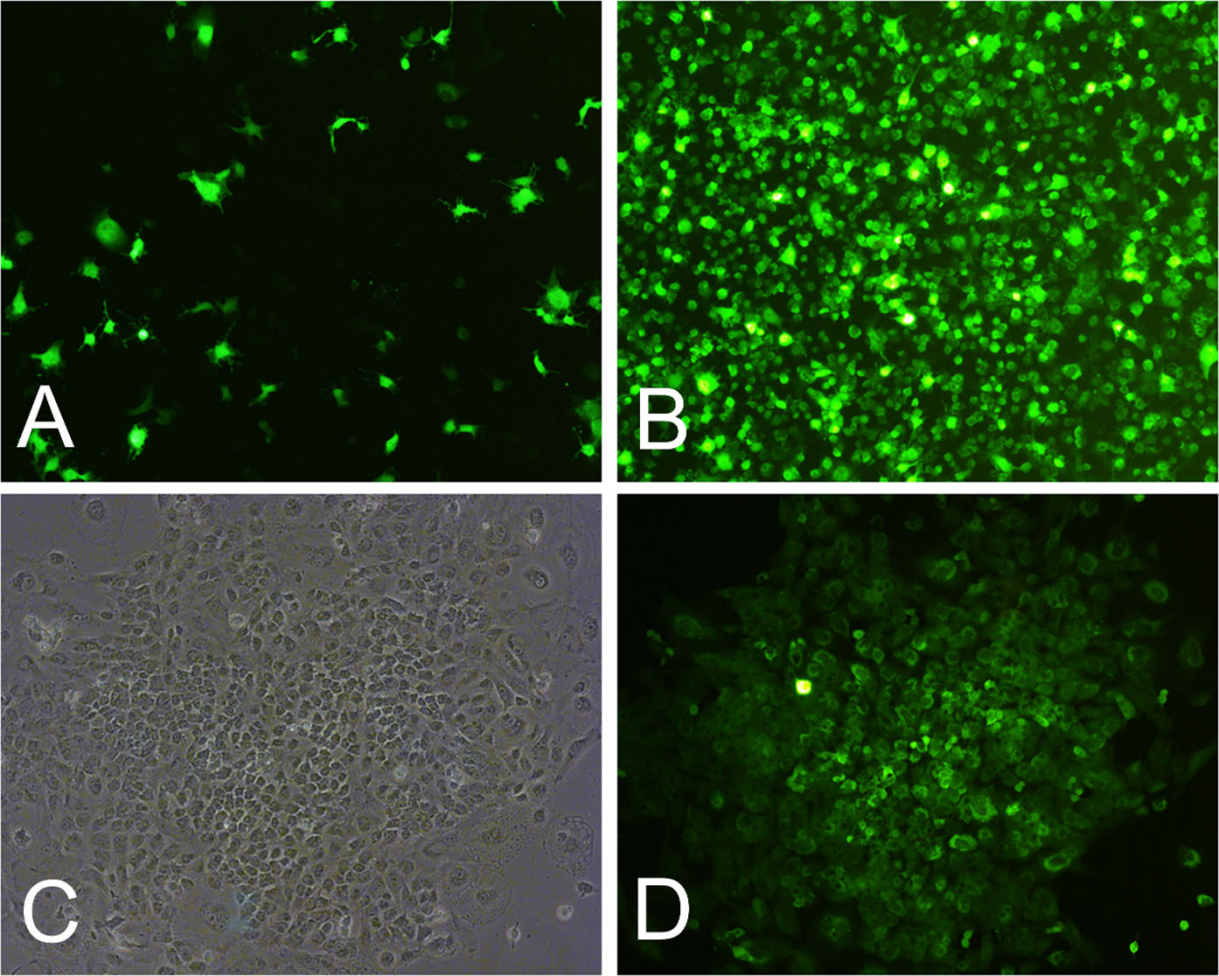
Intestinal epithelial cells infected with lentivirus and screened (× 100). **a** Cells observed by fluorescence microscopy 5 days post-infection. **b** Cells observed by fluorescence microscopy 15 days post-infection. **c** Monoclonal TIEC1s after screening. **d** Monoclonal cells observed by fluorescence microscopy

After infection with lentivirus, cells were continuously observed using fluorescence microscopy. Fifteen days post-infection, the number of cells was higher and the fluorescence was stronger than on the fifth day (Fig. 1a, b), indicating that the ZsGreen gene carried by the vector was expressed.

For isolation of transformed cells, cells were digested and transferred to 96-well plates 15 days post-infection. Three wells were found to contain one cell (Fig. 1c, d) named TIEC1, TIEC2, TIEC3 respectively. As the three clones showed no significant differences in the following experiments, TIEC1 was selected and mainly discussed here. The results of TIEC2 and TIEC3 are shown in supplementary figures.

### Morphology of TIEC1

**Fig. 2:**
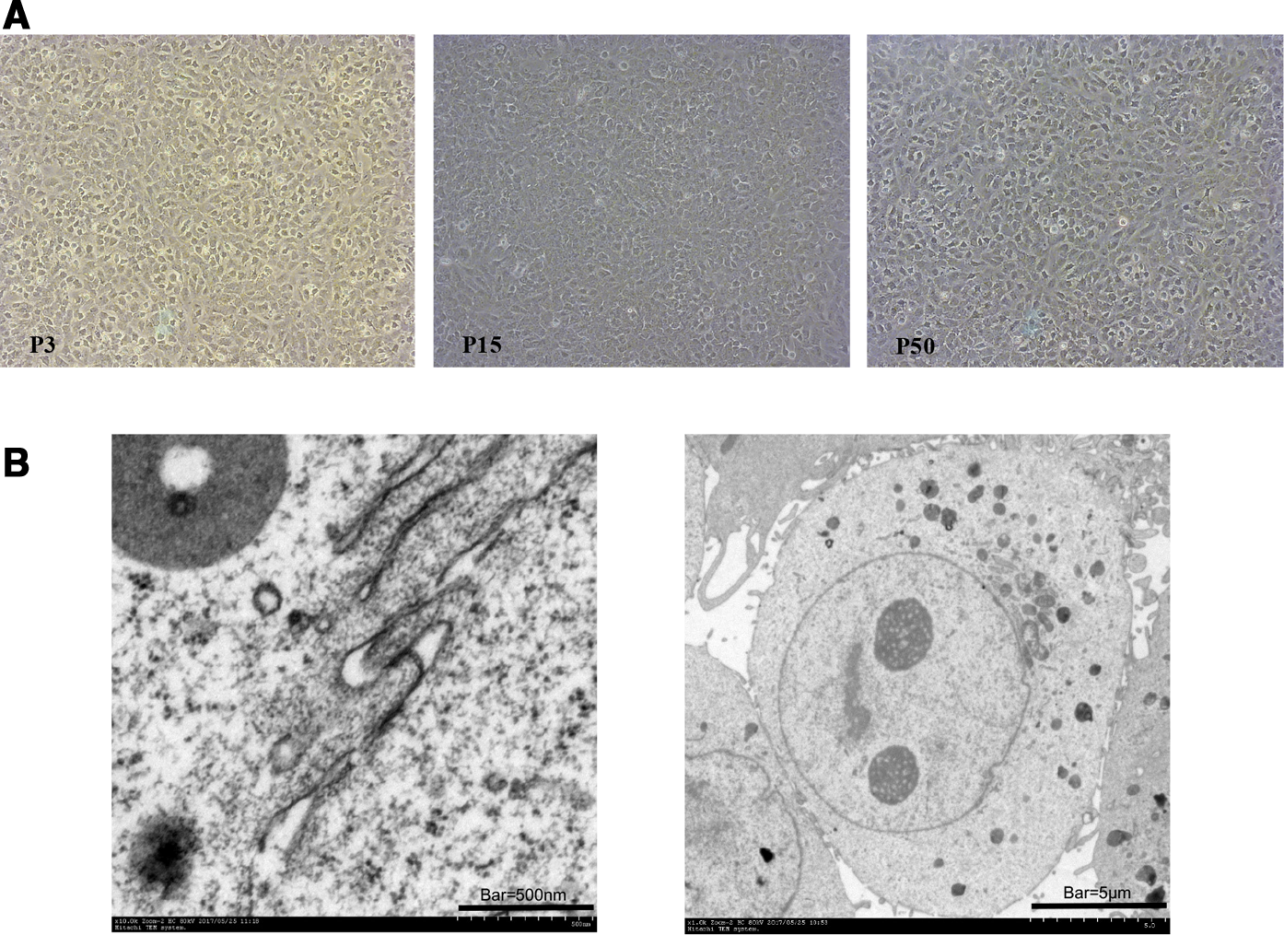
Morphology of intestinal epithelial cells. **a** After 3 generations, pTIECs displayed a typical “cobblestone”-like shape. After 15 generations, TIEC1s became irregular polygons. After 50 generations, TIEC1s became less regular in shape but remained tightly connected (×100). **b** Transmission electron microscopy of TIEC1s after 50 generations. Microvilli and tight junctions are typical features of IECs

Cells obtained by culturing with collagenase XI, neutral protease I, and DTT (Yin et al. 2017) were translucent when observed with an inverted microscope. After incubation for 24 h, cell attachment was assessed. Cells maintained their shape and activity after three passages. However, after 15 passages, TIECs became hexagonal or irregular polygons with translucent cytoplasm, clear cell boundaries, and a typical “cobblestone”-like shape. After 50 passages, cells became less regular in shape but remained tightly connected (Fig. 2a and Supplementary Fig. S1). Transmission electron microscopy used to observe TIEC microstructure revealed a large number of membrane-bound particles, rich ribosome particles, tight junction complexes, and microvilli (Fig. 2b and Supplementary Fig. S2).

### hTERT gene expression analysis by qRT-PCR

To determine whether the *hTERT* gene was integrated into IECs, we used qRT-PCR to detect *hTERT* gene expression in pTIECs and TIECs. Results showed that hTERT mRNA was almost undetectable in pTIECs but was detected in TIECs (Table 1), suggesting that the *hTERT* gene was successfully transferred into IECs and expressed in the transduced cells.

**Table 1:** Quantitative reverse transcription polymerase chain reaction threshold (Ct) values

Sample	pTIEC	TIEC1	TIEC2	TIEC3	*P* value
GAPDH	17.26 ± 0.23	17.17 ± 0.14	17.33 ± 0.10	17.07 ± 0.43	NS
hTERT	> 40^a^	33.10 ± 0.22	32.98 ± 0.20	29.61 ± 6.54	–

Values are given as mean ± SD of three independent experiments

*NS* no statistical significant difference

^a^Value is undetectable in 40 cycles

### TIEC1 expresses IEC-specific molecules

**Fig. 3:**
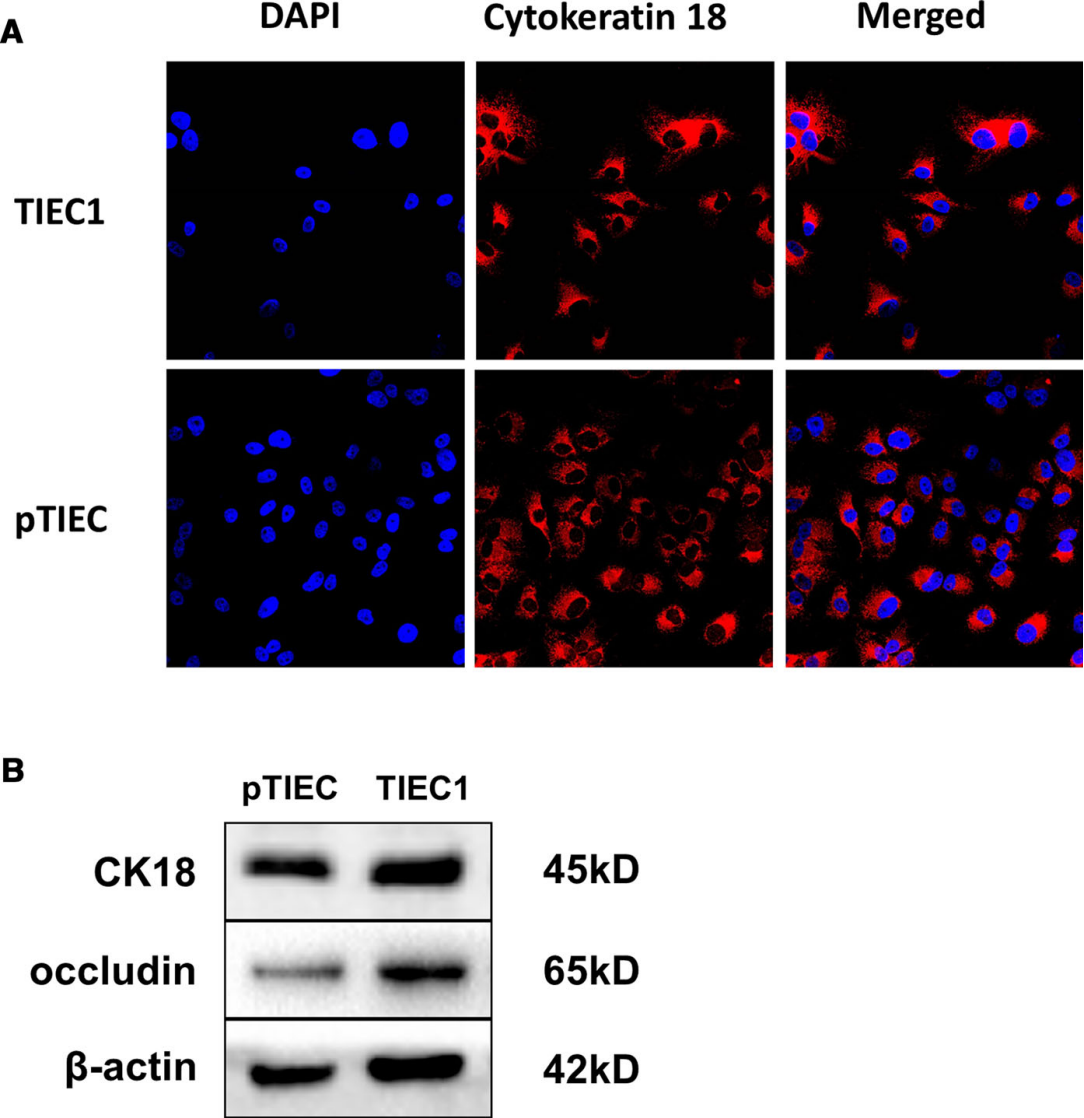
Biochemical characteristics of TIEC1s and pTIECs. **a** Immunofluorescence staining of TIEC1s and pTIECs. The nucleus was stained with DAPI (blue) and cytokeratin 18 was stained with anti-cytokeratin 18 antibodies (red). **b** Detection of proteins by western blot. Cytokeratin 18 and occludin were both expressed in TIEC1s and pTIECs

TIEC1s were characterized using immunofluorescence and western blot assays. Immunofluorescence with anti-cytokeratin 18 antibodies showed that this protein was present in pTIECs, TIEC1s (Fig. 3a) and TIEC2, TIEC3 (Supplementary Fig. S3). Western blot assays with antibodies against cytokeratin 18 and the intracellular protein occludin showed that both proteins were present in TIEC1s, pTIECs (Fig. 3b) and TIEC2, TIEC3 (Supplementary Fig. S4). These results demonstrate that TIEC1s retain the characteristics of pTIECs.

### The proliferative rate of TIECs is higher than that of pTIECs

**Fig. 4:**
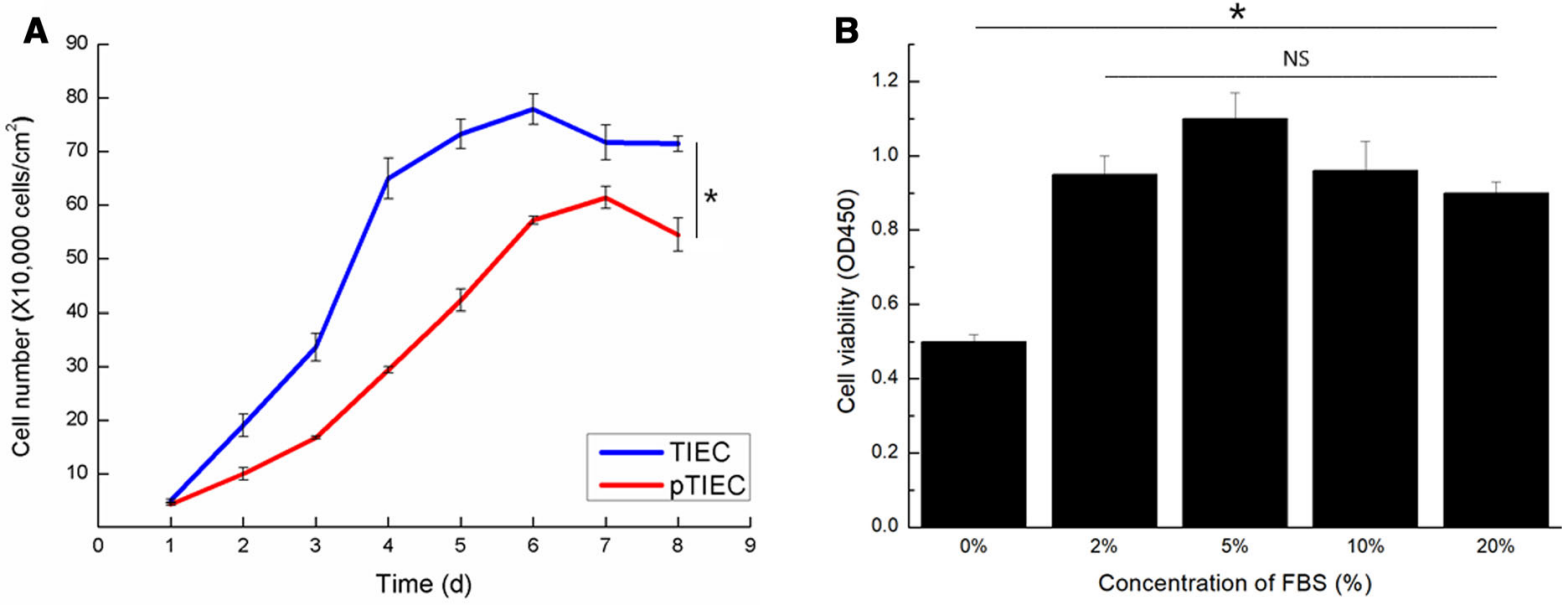
Proliferation assays. Data are presented as mean ± SD of TIEC1, TIEC2 and TIEC3. **a** Growth curve of pTIECs and TIECs. Both growth curves were S-shaped and the proliferation rate of TIECs was higher than that of pTIECs. From d2 to d8, the cell number each day of TIECs was significantly different with that of pTIECs at the 0.05 level. **b** Effect of FBS concentrations on cell viability. The cell proliferation rate in media containing 2–20% FBS was significantly different with that in FBS free media at the 0.05 level. The highest proliferation rate was observed in media containing 5% FBS. *p < 0.05. *NS* not significant

In order to assess the proliferation characteristics of TIECs, growth curves were created and optimal FBS concentration assays were performed. The growth curves of pTIECs and TIECs were both S-shaped (Fig. 4a). However, the proliferation rate of TIECs was higher than that of pTIECs. The number of TIECs peaked after 5–6 days and began to decline after 7 days, whereas the number of pTIECs peaked after 7 days and began to decline after 8 days. These results suggest that TIECs had higher proliferative activity than pTIECs.

Cells were cultured with different concentrations of FBS for 36 h and the optical density at 450 nm was measured with a microplate reader. The results showed that cell proliferation was higher in media containing serum than in serum-free media (Fig. 4b). Although no significant difference in cell proliferation was observed between the different concentrations of FBS, cell proliferation was highest in cultures containing 5% FBS.

### TIECs have the same number of chromosomes as pTIECs

**Fig. 5:**
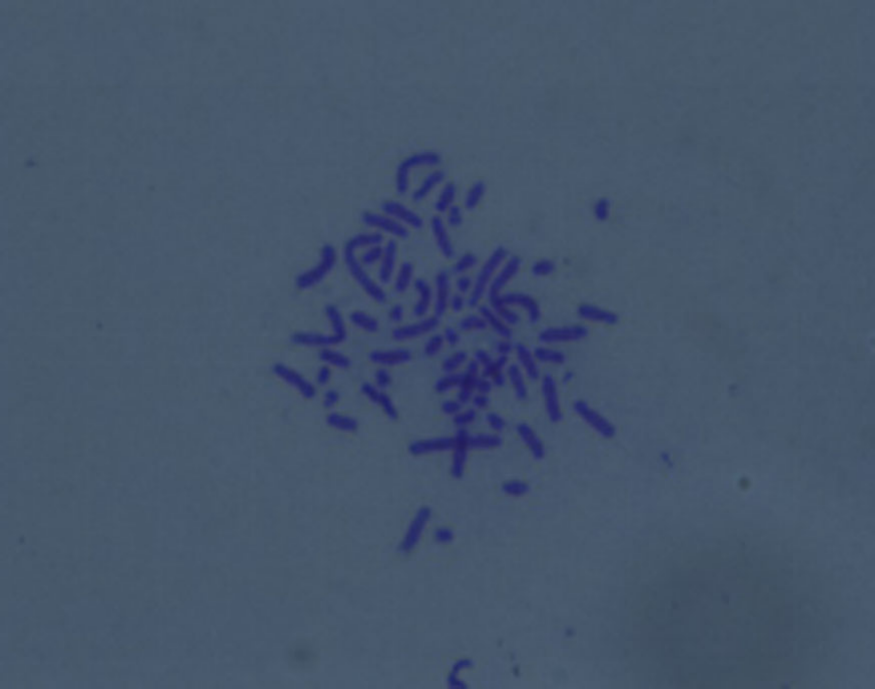
Karyotype analysis of TIEC1s (× 1000). TIEC1s were diploid cells maintaining the same number of chromosomes (62) as pTIECs

TIEC1s contained 62 chromosomes (Fig. 5), consistent with both pTIECs and the previously reported number of tree shrew chromosomes (Ma 2016). Similarly, TIEC1s were diploid cells, demonstrating that chromosome morphology was normal in these cells. The data of TIEC2s and TIEC3s were demonstrated in Supplementary Fig. S5.

## Discussion

In this study, we established an immortalized tree shrew IEC cell line by lentivirus-mediated gene transfer of the exogenous *hTERT* gene. Previously, several strategies for immortalization were reported. Ma (2016) transfected the SV40Tag gene into primary tree shrew hepatocytes to establish immortalized cells. However, follow-up experiments showed that telomere length shortened as the number of passages increased. Hurlin et al. (1991) established a cell line by transfection with the HPV16 gene, but this caused a change in the genotype of the cells. The discovery of hTERT was a significant step for studies of cell proliferation, immortalization, and tumor transformation (Klingelhut 1999), as expression of the *hTERT* gene protects cells from telomerase activity. Maeda et al. (2005) successfully established five immortalized human uterine epithelial cell lines by transfecting the *hTERT* gene into uterine epithelial cells. This allowed for the original diploid karyotype to be maintained and did not cause a malignant phenotype. Similarly, an immortalized human liver cell line was established by Reid et al. (2009) by transfection of a recombinant retrovirus expressing the *hTERT* gene. These studies showed that hTERT allows primary cells to become immortalized while retaining their original cell characteristics and functions.

Previously, we transfected the exogenous *hTERT* gene using liposomes because their immunogenicity is lower than that of viral vectors, the size of the target gene does not affect transfection efficiency, experiments using liposomes take less time than packaging of viral vectors, and experimental material is more easily obtained from liposome transfection. However, we were unable to obtain transfected cells in these experiments. Therefore, we turned to lentivirus-mediated transduction of the exogenous *hTERT* gene. We also introduced a reporter gene, ZsGreen, in the lentivirus package to easily detect successfully transduced cells.

Interestingly, when we added puromycin 72 h post-infection, we did not obtain any positive clones. However, after optimization, we developed a protocol in which cells were cultured in a manner similar to primary cells for 15 days post-infection, after which positive clones could be obtained by adding puromycin. We did find that the growth rate of cells was significantly reduced after 15 days due to the addition of puromycin. Two generations after infection, uninfected cells were still present. However, as the number of passages increased, the number of uninfected cells was reduced until only transduced cells remained. At this point, the cell growth rate reached its minimum.

During our experiments, irregular cell shapes were observed and the boundaries between cells became blurred in TIEC cells. Initially, phenol red caused the cell culture media to remain red, indicating a very low cell growth rate. However, when the media were replaced every other day, the cells began to appear pebble-like and the growth rate became higher than that of primary cells. At this point, the transduced cells began to grow steadily.

Cytokeratin is a keratin intermediate filament found in the epithelial cell cytoskeleton and is often used as a marker to identify epithelial cells. Cytokeratin 18 is an epithelial cell-specific molecule that is often used to specifically identify IECs (Perreault and Beaulieu 1998; Rusu et al. 2005; Su et al. 2013). In the present study, immunofluorescence and western blot experiments showed that cytokeratin 18 was present in TIECs. Similarly, occludin is an important protein for the stability and barrier function of tight junctions (Simon-Assmann et al. 2007). IECs have been generated from undifferentiated cells in the villi of the small intestine. IECs express tight junction proteins that undifferentiated basal cells do not express that allow them to form a physical barrier in the intestinal epithelium. Our western blot experiments showed that occludin was present in TIECs, further demonstrating that TIECs are epithelial cells (Parthasarathy and Mansfield 2009). In addition, electron microscopy showed that TIECs have microvilli on their surfaces and that the cells are closely connected. These results indicate that TIECs are differentiated into villi cells and that they maintain the characteristics of epithelial cells.

The growth rate of TIECs in 5% FBS was higher than that of pTIECs. Similar to primary cell cultures, in which 2% FBS was added to inhibit the growth of fibroblasts, transduced cells were found to proliferate poorly in the absence of FBS. Chromosome analysis showed that TIECs had 62 chromosomes, the same number that tree shrews have under normal physiological conditions. Therefore, the immortalized cell line obtained by *hTERT* gene transfer can be used as a method to immortalize IECs while maintaining their normal physiological properties. The *hTERT* gene was successfully transferred into tree shrew IECs using lentivirus and long-term stable expression of the gene was achieved. The results of qRT-PCR experiments showed that hTERT mRNA was detectable in TIECs but not pTIECs, which is in line with the results of Wang et al. (2014). In addition, results showed that telomerase activity was activated by transgenic hTERT. These transduced TIECs could be passaged in vitro for more than 50 generations owing to the successful transfer of the *hTERT* gene. Therefore, we conclude that *hTERT* gene transfer allows for the immortalization of tree shrew IECs and is a new alternative cell model for research.

## Electronic supplementary material

Below is the link to the electronic supplementary material.

Supplementary Fig. S1 Morphology of TIEC2s and TIEC3s at passages 50 (100 ×) (TIFF 1259 kb)

Supplementary Fig. S2 Transmission electron microscopy of TIEC2s and TIEC3s after 50 generations (TIFF 869 kb)

Supplementary Fig. S3 Immunofluorescence staining of TIEC2s and TIEC3s. The nucleus was stained with DAPI (blue) and cytokeratin 18 was stained with anti-cytokeratin 18 antibodies (red) (TIFF 633 kb)

Supplementary Fig. S4 Detection of proteins by western blot (TIFF 136 kb)

Supplementary Fig. S5 Karyotype analysis of TIEC1s (1000 ×) (TIFF 645 kb)
