# Supplementary figures and images for: Establishment of an immortalized intestinal epithelial cell line from tree shrews by lentivirus-mediated hTERT gene transduction
Source: Cytotechnology. 2019 Jan 2;71(1):107–16. doi: 10.1007/s10616-018-0270-0 (PMC6368523; doi:10.1007/s10616-018-0270-0)

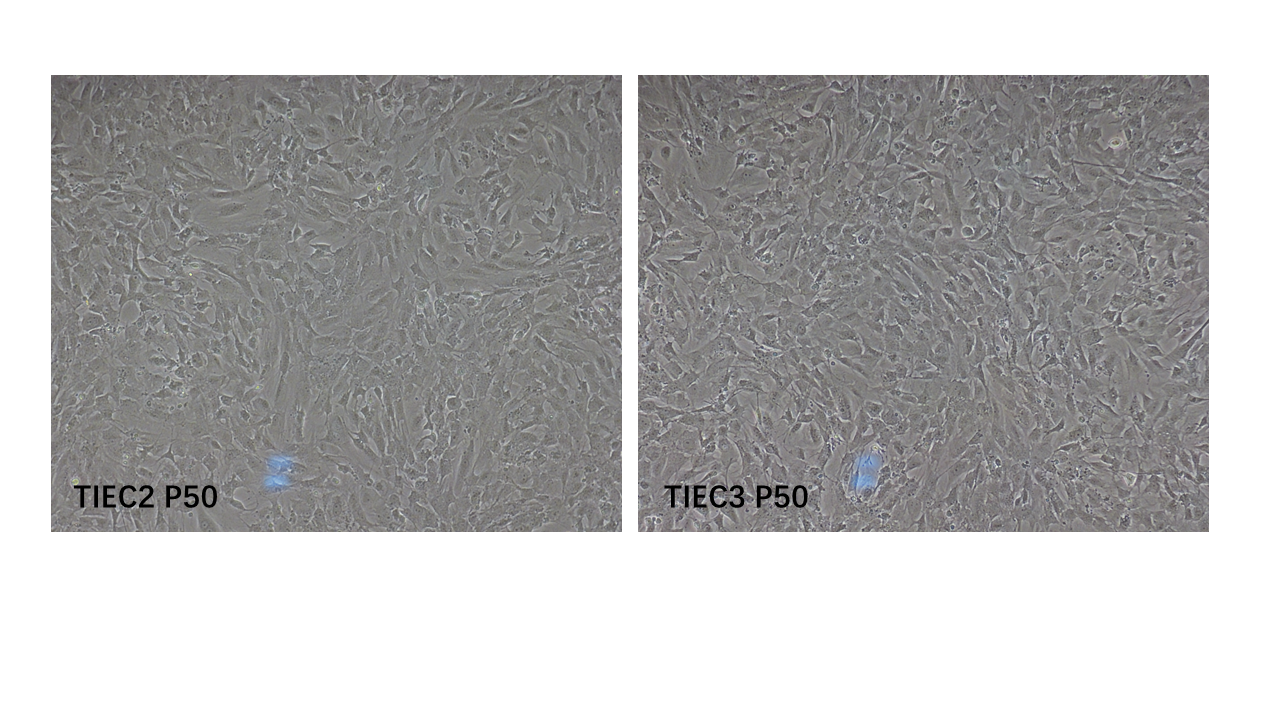

Supplement: Supplementary file 1 — Supplementary Fig. S1 Morphology of TIEC2s and TIEC3s at passages 50 (100 ×) (TIFF 1259 kb) [file 10616_2018_270_MOESM1_ESM.tif]

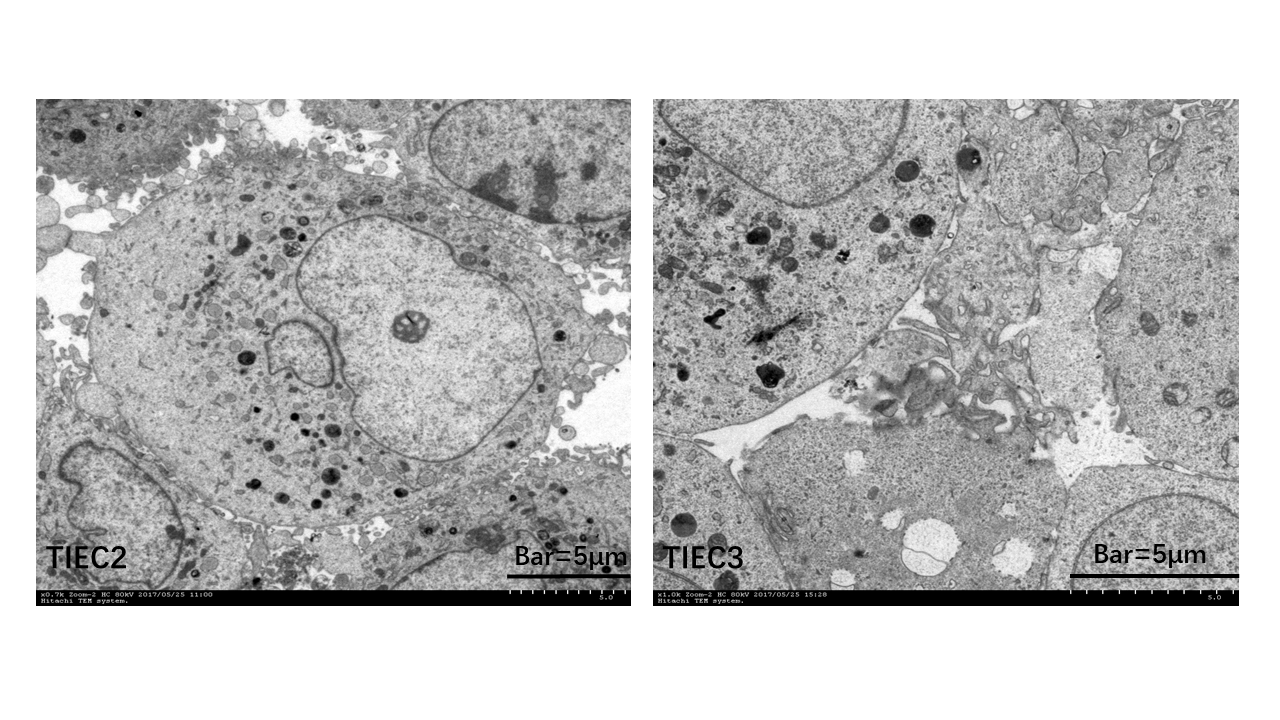

Supplement: Supplementary file 2 — Supplementary Fig. S2 Transmission electron microscopy of TIEC2s and TIEC3s after 50 generations (TIFF 869 kb) [file 10616_2018_270_MOESM2_ESM.tif]

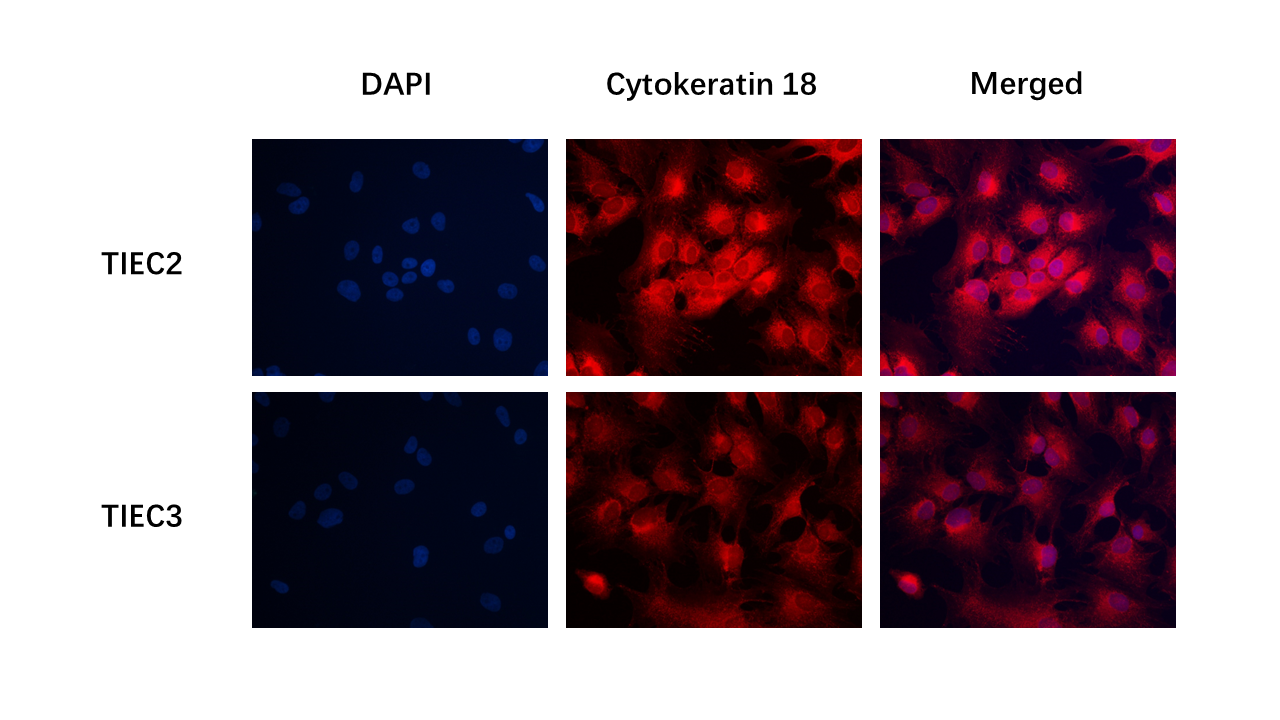

Supplement: Supplementary file 3 — Supplementary Fig. S3 Immunofluorescence staining of TIEC2s and TIEC3s. The nucleus was stained with DAPI (blue) and cytokeratin 18 was stained with anti-cytokeratin 18 antibodies (red) (TIFF 633 kb) [file 10616_2018_270_MOESM3_ESM.tif]

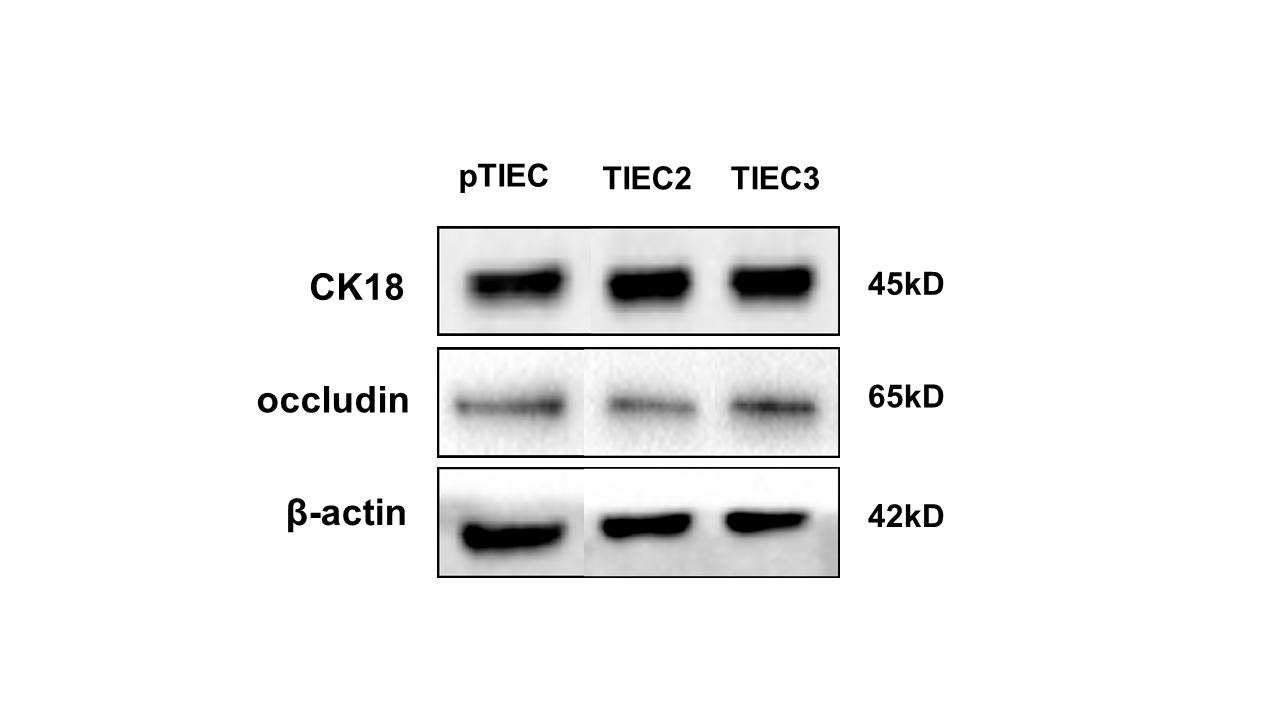

Supplement: Supplementary file 4 — Supplementary Fig. S4 Detection of proteins by western blot (TIFF 136 kb) [file 10616_2018_270_MOESM4_ESM.tif]

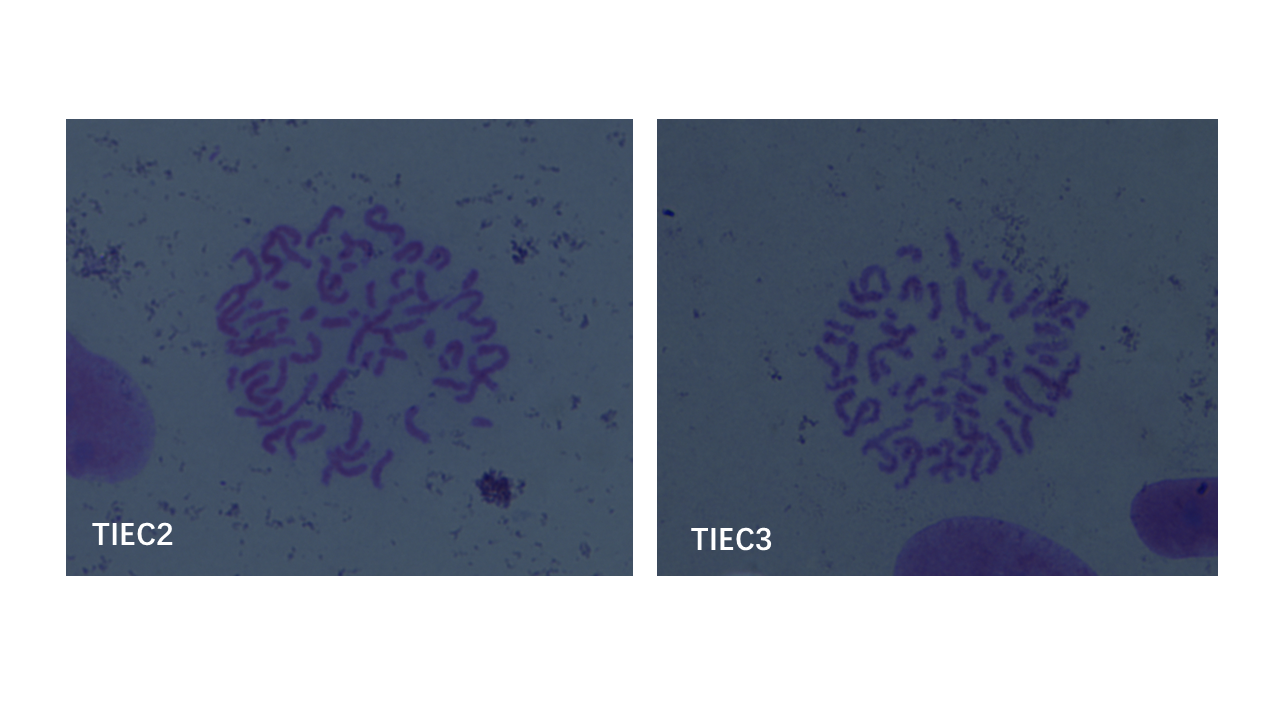

Supplement: Supplementary file 5 — Supplementary Fig. S5 Karyotype analysis of TIEC1s (1000 ×) (TIFF 645 kb) [file 10616_2018_270_MOESM5_ESM.tif]
